# Psychological Assessment of Illness Denial in Medical Settings: A Critical Review

**DOI:** 10.1002/cpp.70240

**Published:** 2026-02-16

**Authors:** Danilo Carrozzino, Sara Gostoli

**Affiliations:** ^1^ Department of Psychology “Renzo Canestrari” University of Bologna Bologna Italy

**Keywords:** assessment, clinical utility, clinimetrics, illness denial, medical disorders, staging

## Abstract

This is the first critical review to assess the clinimetric properties, particularly the sensitivity (i.e., the ability to detect changes in clinical trials and yield clinical distinctions that may demarcate major prognostic and therapeutic differences), and to determine the clinical utility of instruments of illness denial in patients with medical disorders. A comprehensive search of the literature was performed on several databases. Patient‐reported outcome measures and clinician‐rated instruments of illness denial were identified and analysed. The findings indicate that the Acceptance and Action Diabetes Questionnaire and the Denial of Illness Scale can be used as screening tools to early detect diabetes and stroke patients at increased risk for clinical complications. The Diagnostic Criteria for Psychosomatic Research and the Levine Denial of Illness Scale are transdiagnostic instruments that are particularly suitable to identify the maladaptive manifestations of illness denial and its affective, behavioural and cognitive components. The Illness Denial Questionnaire, particularly the eight‐item version of the instrument, is another transdiagnostic measure that may be better suited for the evaluation of illness denial severity. The Denial of Cancer Interview can be used for a longitudinal monitoring of illness denial, whereas the Hackett–Cassem Denial Scale is recommended to assess the degree of illness denial in individuals with cardiac disorders. The use of instruments such as the Cardiac Denial of Impact Scale, the Denial Questionnaire and the Havik and Mæland Denial Scale, which were found to have considerable prognostic utility, may help patients promptly recognize and respond to life‐threatening diseases.

## Summary

- Instruments of illness denial may facilitate early detection of clinical complications.
- Assessment of illness denial may have considerable prognostic value.
- Certain instruments of illness denial may have transdiagnostic utility.
- Some of the existing instruments can be used to assess the severity of illness denial and its impact on treatment outcomes and adherence.
- Certain instruments can be used to assess the rate of progression of illness denial.

## 1 Introduction

In the emerging framework of lifestyle medicine (Fava 2023), the role of illness denial (Goldbeck 1997) has attracted substantial attention. The concept of illness denial was originally introduced to describe patients with medical disorders who do not accept their diagnosis or appear oblivious to it or detached, minimize the implications of their illness, tend to delay seeking medical advice and refuse or comply poorly with treatment (Goldbeck 1997). Several authors recently advocated the need to incorporate illness denial into current diagnostic classification systems, and its early detection has also been regarded as a major healthcare challenge (Fricchione 2023; Horwitz and Cullen 2023; Patierno et al. 2023; Wise 2023).

Methods of assessment of illness denial have ranged widely over the years but have commonly involved the use of clinical judgement (Dean and Surtees 1989; Gentry et al. 1972; Greer et al. 1979). The presence or absence of denial was mainly inferred from answers to just a single question, an evaluation method that was, however, found to be subject to a high degree of inter‐rater variability (Croog et al. 1971; Gentry et al. 1972; Rogentine et al. 1979).

Hackett and Cassem (1974) were the first to attempt to assess illness denial in a quantitative way. They developed the Hackett–Cassem Denial Scale (HCDS), a semi‐structured interview, which was originally administered to patients with a diagnosis of suspected or confirmed myocardial infarction (Hackett and Cassem 1974). The introduction of this interview for the identification of illness denial has considerably decreased the variance among raters but the scale has been criticized for measuring aspects that were not always strictly related to illness denial (Beisser 1979; Havik and Mæland 1986). Fowers (1992) also noted that Hackett and Cassem relied on the psychometric assumption of homogeneity of components in the initial development of their interview, thus regarding illness denial as a homogeneous clinical entity.

This notion of illness denial has been questioned given the multidimensional features and various manifestations of denial, which did not necessarily involve negative outcomes and maladaptive responses to illness but also health‐promoting attitudes and behaviours (Douglas and Druss 1987; Goldbeck 1997; Shelp and Perl 1985; Wheeler and Lord 1999).

Further, the psychometric principle of homogeneity of components is no longer required in current assessment strategies, particularly in clinimetrics, the science of clinical measurements (Fava et al. 2012), where multidimensional indices rather than homogeneous measures are expected to be used for assessing the distinctive characteristics of the complex clinical conditions under evaluation (Carrozzino et al. 2021).

### 1.1 From Classical Psychometrics to Clinimetrics

In classical psychometrics, a theory grounded in the educational and social areas, priority has been given to statistical coherence of rating scales, usually consisting of multiple items, which are primarily selected and tested for reliability and validity mainly based on coefficients (e.g., Cronbach's alpha) emerging from correlation analyses that are often of statistical but not always of clinical significance (Bech 2004; Carrozzino et al. 2021). In one of the first studies addressing the most significant conceptual and methodological differences between psychometrics and clinimetrics, Wright and Feinstein (1992) noted that because the items in a psychometric scale are usually combined according to high statistical correlations, they may display poor clinical validity despite the impressive mathematical associations. In the clinimetric approach, the psychometric criterion of statistical coherence is supplemented by the clinimetric principle of clinical coherence based on the assumption that psychometric analyses must not be an alternative to clinical thinking and reasoning (Bech 2004). Assessment instruments may display various forms of clinical coherence that can be adequately evaluated based on clinimetric criteria (Bech 2004; Carrozzino et al. 2021). The following are different but interrelated forms of clinical coherence that an assessment instrument may entail: inter‐clinician reliability (i.e., the level of agreement or concordance between two or more clinical investigators who independently evaluate the same clinical phenomenon using the same clinical interview); clinimetric sensitivity (i.e., the ability of an instrument [1] to improve the detection of clinically relevant changes in drug and psychotherapy trials, [2] to differentiate patients from healthy controls, [3] to distinguish between different groups of patients suffering from the same illness, [4] to discriminate between an active treatment and placebo, [5] to differentiate between wanted and unwanted effects of treatments); clinical validity (i.e., the ability of an instrument to reflect the clinician's evaluation of severity of the clinical condition under assessment); construct validity (i.e., the extent to which each item on an assessment instrument reflects one single dimension of severity of the same underlying clinical condition); concurrent validity (i.e., the degree of concordance between two instruments that are supposed to measure the same clinical condition); incremental validity (i.e., the distinctive contribution or incremental increase in the predictive accuracy of a particular assessment instrument); and predictive validity, which refers to the ability of an instrument to predict clinical outcomes or stratify patients into distinctively different prognostic groups (Bech 2004; Carrozzino et al. 2021).

### 1.2 Instruments of Illness Denial

Over the years, many measures have been introduced to assess illness denial (Fowers 1992; Levine et al. 1987; Starkstein et al. 1993), but classical psychometric principles mainly guided the construction and validation of most of the available instruments (Rossi Ferrario et al. 2019; Vos et al. 2007). It is, however, important to note that also when developed according to psychometric criteria, clinician‐rated instruments and patient‐reported outcome measures (PROMs), any reports coming directly from patients about how they function or feel in relation to a health condition and its therapy (Carrozzino et al. 2021), may have a certain clinical utility (Carrozzino et al. 2020; Carrozzino et al. 2023). A systematic review (Patierno et al. 2023) was recently published to determine the impact of illness denial in patients with several medical conditions, but data regarding the clinical utility of available instruments of illness denial have not been provided. Given the lack of studies providing clear indications regarding the assessment of illness denial in medical settings, there is the need for a review of studies to describe the clinimetric properties and clarify the clinical applications of existing instruments of illness denial.

### 1.3 Aims

This is the first critical review, which is aimed at identifying clinician‐rated instruments and PROMs, which best display the clinimetric properties of reliability, sensitivity and validity in the clinical process of assessment of illness denial in patients with different medical disorders. The present critical review is not limited to a summary of findings but will determine the clinical utility of existing instruments of illness denial and provide practical indications for their current and potential use in clinical research and practice with medical patients.

## 2 Methods

### 2.1 Search Strategy

A comprehensive search of the literature was conducted using PubMed, Scopus and Web of Science. Each database was searched from inception to July 2025. Reference lists of the included articles were also examined for further studies not yet identified. The MeSH terms were ‘denial’, ‘disease’, ‘disorder’ and ‘illness’. They were combined using ‘AND’ and ‘OR’ as Boolean operators.

### 2.2 Eligibility Criteria

To be included in this critical review, studies had to meet the following eligibility criteria: (1) English‐language article published in a peer‐reviewed journal; (2) the full text of the article was available online or after request to the authors; (3) the article was an original research paper (e.g., a validation study or a clinical trial) in which illness denial (Goldbeck 1997) was assessed using a clinician‐rated instrument or a self‐reported measure; (4) the study provided data regarding the clinimetric properties (Bech 2012; Carrozzino et al. 2021) of instruments of illness denial in adult patients (i.e., older than 18 years) with medical disorders.

Single case reports, general population studies or those where qualitative data were only available (e.g., commentaries, opinion articles) and studies that only evaluated the psychometric properties (for instance, those in which the reliability and validity of measures of illness denial were established using Cronbach's alpha coefficients and exploratory/confirmatory factor analyses) were excluded. Studies conceiving illness denial not as a distinct clinical phenomenon but as the result of a psychiatric or neurodegenerative disorder characterized by lack of insight or anosognosia were excluded. There were no restrictions regarding the year of publication and the study design.

### 2.3 Study Selection

Two authors (D.C. and S.G.) independently conducted the search of the literature, screened titles and abstracts for inclusion, assessed the full text of articles appearing potentially relevant, selected studies meeting the inclusion criteria and extracted data that were concerned with the clinimetric properties of instruments of illness denial. In case of disagreement, a consensus was reached through discussion.

### 2.4 Data Extraction and Synthesis

Data, which were concerned with clinimetric properties (Bech 2012; Carrozzino et al. 2021) of inter‐rater reliability, sensitivity (including both discriminant ability and sensitivity to change), clinical, concurrent, construct, incremental and predictive validity were extracted, analysed and critically discussed to determine the current and potential clinical applications of existing instruments of illness denial.

### 2.5 Methodological Quality

Methodological quality of studies included in the present critical review was assessed using the Consensus‐based Standards for the selection of health Measurement Instruments (COSMIN) checklist (Mokkink et al. 2010; Terwee et al. 2012). However, adaptations were needed not only because the COSMIN checklist was originally developed for use in studies on the quality of PROMs but also given that these guidelines mainly apply to the evaluation of overall methodological quality of studies on the psychometric properties of instruments (Mokkink et al. 2010; Terwee et al. 2012). Because this is a critical review on the clinimetric properties of existing PROMs and clinician‐rated instruments of illness denial, clinimetric criteria (Bech 2012; Carrozzino et al. 2021) and guidelines (Bech 2004; Fava et al. 2012; Feinstein 1982) were also followed to determine the methodological quality of included studies. Methodological quality of each study was scored on a 4‐point rating system ranging from *very good* to *inadequate*. Methodological quality is rated as *very good* when there is clear evidence that the standard for the analysis of the clinimetric property under evaluation is met (Mokkink et al. 2010; Terwee et al. 2012). Quality is rated as *adequate* when it is assumable, although not explicitly described, that the standard is met. The *doubtful* rating is generally given when it is unclear if the standard is met, whereas the quality is scored as *inadequate* when there is clear evidence that the standard is not met (Mokkink et al. 2010; Terwee et al. 2012). Levels of evidence regarding the overall quality of clinimetric properties for each instrument of illness denial have also been determined based on COSMIN criteria (Mokkink et al. 2010; Terwee et al. 2012). The evidence level is rated as *strong* when consistent findings are reported in multiple studies of *adequate* methodological quality or in one study of *very good* methodological quality. The evidence level is scored as *moderate* when consistent findings are shown in multiple studies of *doubtful* methodological quality or in one study of *adequate* methodological quality. The evidence level is rated as *limited* when findings are reported in one study of *doubtful* methodological quality. The evidence level is rated as *conflicting* when contrasting results are obtained. Finally, the evidence level is scored as *unknown* when findings are shown in only studies of *inadequate* methodological quality (Mokkink et al. 2010; Terwee et al. 2012). Two reviewers (D.C. and S.G.) independently evaluated the methodological quality of included studies. In case of disagreement, discrepancies between authors were resolved through discussion.

## 3 Results

The initial search of the literature yielded a total of 201 articles on illness denial, of which 129 were removed based on exclusion criteria. Of these, 76 articles (59%) were not selected given that only a classical psychometric approach was used to assess the reliability and validity of existing PROMs and clinician‐rated instruments of illness denial. The remaining 53 (41%) were excluded because they were clinical studies, in which data regarding the clinimetric properties of instruments were not reported. A total of 72 studies met the inclusion criteria and were discussed in this critical review. The list of included studies and their main characteristics are presented in the Appendix S1 (Table A.1). Twelve instruments of illness denial were identified and analysed. Seven of them were PROMs (Carver et al. 1989; Fowers 1992; Gregg et al. 2007; Havik and Mæland 1986; Pilowsky and Spence 1975; Rossi Ferrario et al. 2017; Trijsburg et al. 1987), whereas the other five clinician‐rated instruments (Fava et al. 1995; Hackett and Cassem 1974; Levine et al. 1987; Starkstein et al. 1993; Vos et al. 2007). A full description of each instrument of illness denial is provided in Table 1, whereas clinimetric properties and clinical utility of existing measures are summarized in Table 2. Data regarding the methodological quality of included studies are reported in Table 3, while levels of evidence for the overall quality of each clinimetric property are shown in Table 4. Methodological quality for the majority of included studies (*n* = 57; 79%) was adequate or very good (Table 3). Regarding the overall quality of each clinimetric property, strong evidence was found, particularly for the clinimetric sensitivity of PROMs and clinician‐rated instruments of illness denial (Table 4).

**TABLE 1:** Instruments of illness denial.

Measure	Description
	Patient‐reported outcome measures
Acceptance and Action Diabetes Questionnaire (AADQ)	The AADQ is a 11‐item patient‐reported outcome measure that was originally developed to assess acceptance of diabetes‐related thoughts and feelings and the degree to which they interfere with valued actions. The AADQ also includes several items (e.g., ‘I often deny to myself what diabetes can do to my body’ and ‘I avoid thinking about what diabetes can do to me’) that reflect an experience of illness denial. Each item of the AADQ is scored on a 7‐point Likert scale with responses ranging from 1 (i.e., *never true*) to 7 (i.e., *always true*)
Cardiac Denial of Impact Scale (CDIS)	The CDIS is an eight‐item patient‐reported outcome measure that was developed to assess the tendency to deny the emotional impact of cardiac disease. Items (e.g., ‘I was not at all afraid when I learned that I had a heart attack’) are scored on a 5‐point Likert scale ranging from *strongly agree* to *strongly disagree*. A cut‐off score of ≥ 25 indicates cardiac denial
Coping Orientations to Problems Experienced (COPE)	The COPE is a self‐rated inventory of coping responses that range from problem‐focused strategies (e.g., active coping) to the use of avoidance. This measure includes a denial subscale, which evaluates the individual tendency or attitude of refusing to believe that the problem (e.g., illness) exists. Items of the COPE (e.g., ‘I've been refusing to believe that it has happened’) are rated on a 4‐point Likert scale ranging from 1 (i.e., *I have not been doing this at all*) to 4 (i.e., *I have been doing this a lot*)
Denial Questionnaire (DQ)	The DQ is a patient‐reported outcome measure that also contains a scale completed by a significant other, usually a partner, caregiver or family member. Both the patient and partner are asked to assess the patient's behaviour prior to the onset of acute myocardial infarction. In case of denial, the significant other gives a more negative opinion about the patient's behaviour as compared to the patient's own judgement
Havik and Mæland Denial Scale (HMDS)	The HMDS is an eight‐item self‐reported questionnaire that was developed to evaluate illness denial in patients with myocardial infarction. Items of the HMDS (e.g., ‘deep inside, I'm not really convinced that I have had a heart attack’) are rated on a 5‐point Likert ranging from *completely disagree* to *completely agree*, with higher scores indicating a greater denial of myocardial infarction
Illness Behaviour Questionnaire (IBQ)	The IBQ is a 52‐item self‐reported questionnaire that has been developed to assess patients' attitudes and feelings about their illness. The IBQ includes a denial subscale. Each item of the IBQ (e.g., ‘except for your illness, do you have any problems in your life?’) is scored on a dichotomous response scale (i.e., yes or no), with higher scores indicating maladaptive ways of perceiving, evaluating or acting upon one's state of health
Illness Denial Questionnaire (IDQ)	The IDQ is a 24‐item patient‐reported outcome measure that was developed to assess three dimensions of illness denial: denial of negative emotions (e.g., ‘thinking about this disorder/disease leaves me quite indifferent’), resistance to change (e.g., ‘the treatments [medications, exercises or others] do not in fact change my life’) and conscious avoidance (e.g., ‘the best way to cope with this disorder/disease is to not think about it’). Each item is rated on a dichotomous (false = 0; true = 1) response format, with higher scores indicating more severe illness denial
	Clinician‐rated instruments
Denial of Illness Scale (DIS)	The DIS is a 10‐item clinician‐rated measure that was originally designed to detect the presence of illness denial and assess its severity in stroke patients. The DIS total score can range from 0 to 16, with higher scores indicating greater intensity of illness denial
Diagnostic Criteria for Psychosomatic Research (DCPR)	The DCPR were introduced by a group of international experts who translated psychosocial variables deriving from psychosomatic research into operational tools. An updated version of DCPR was published in 2017 and is available online. The DCPR cover a total of 14 psychosomatic syndromes, including a diagnostic module on illness denial. The DCPR were designed to be used as a semi‐structured interview with questions that may be modified and reworded if circumstances necessitate. Items of the semi‐structured interview for DCPR are scored through a yes/no response format
Hackett–Cassem Denial Scale (HCDS)	The HCDS is a 31‐item clinician‐rated instrument for the assessment of illness denial severity. The total score of the HCDS ranges from 0 to 64, with higher scores indicating greater denial behaviour
Levine Denial of Illness Scale (LDIS)	The LDIS is a 24‐item semi‐structured interview that can be used to assess the affective, behavioural and cognitive manifestations of illness denial. Each question is rated on a 7‐point Likert scale, with higher scores indicating that the patient has no interest in knowing about her/his illness or that she/he tried to avoid discussing about it. The interviewer may modify the wording of items, add or drop certain questions (e.g., ‘has your illness been explained to you?’), depending upon completeness of the patient's answers
Denial of Cancer Interview (DCI)	The DCI is a clinician‐rated instrument that was developed to assess illness denial in cancer patients. The DCI consists of three parts. The first section includes a nine‐item semi‐structured interview, the second part refers to the clinician's impression of the patient's type of denial (e.g., denial of affect or denial of impact), whereas the last part is concerned with the clinician's impression of the overall level of illness denial. Items of the DCI (e.g., ‘are there times when you do not think about your illness?’) were generated based on clinical observations and consultations with lung cancer patients

**TABLE 2:** Clinimetric properties and clinical utility of existing instruments of illness denial.

Instrument	Response format	Clinimetric properties	Clinical utility
	**Patient‐reported outcome measures (PROMs)**		
Acceptance and Action Diabetes Questionnaire	Likert scale	Clinimetric sensitivity Concurrent and construct validity	Screening Staging
Cardiac Denial of Impact Scale	Likert scale	Clinimetric sensitivity Concurrent and incremental validity	Prognostic
Coping Orientations to Problems Experienced	Likert scale	Clinimetric sensitivity Incremental and predictive validity	Prognostic
Denial Questionnaire	Dichotomous scale	Clinimetric sensitivity	Prognostic
Havik and Mæland Denial Scale	Likert scale	Concurrent and incremental validity	Prognostic
Illness Behaviour Questionnaire	Dichotomous scale	Clinimetric sensitivity	Prognostic
Illness Denial Questionnaire	Dichotomous scale	Clinimetric sensitivity Concurrent, construct and predictive validity	Staging
	**Clinician‐rated instruments**		
Denial of Illness Scale	Likert scale	Clinimetric sensitivity	Screening
Diagnostic Criteria for Psychosomatic Research	Dichotomous scale	Clinimetric sensitivity	Diagnostic
Hackett–Cassem Denial Scale	Likert scale	Inter‐rater reliability Clinimetric sensitivity Clinical and concurrent validity	Staging
Levine Denial of Illness Scale	Likert scale	Inter‐rater reliability Clinimetric sensitivity Concurrent and incremental validity	Diagnostic
Denial of Cancer Interview	Likert scale	Inter‐rater reliability Clinimetric sensitivity	Staging

**TABLE 3:** Methodological quality of studies included in the critical review.

Studies	Instruments of illness denial	Clinimetric properties						
		Inter‐rater reliability	Sensitivity	Validity				
				Clinical	Concurrent	Construct	Incremental	Predictive
Adams et al. (2001)	Illness Behaviour Questionnaire	—	Adequate	—	—	—	—	—
Battaglia et al. (2018)	Diagnostic Criteria for Psychosomatic Research	—	Doubtful	—	—	—	—	—
Bleeker et al. (1995)	Denial Questionnaire	—	Adequate	—	—	—	—	—
Campbell et al. (1995)	Illness Behaviour Questionnaire	—	Very good	—	—	—	—	—
Carver et al. (1993)	Coping Orientations to Problems Experienced	—	Adequate	—	—	—	Adequate	—
Cooke et al. (2003)	Illness Behaviour Questionnaire	—	Adequate	—	—	—	—	—
Cvitanović et al. (2020)	Coping Orientations to Problems Experienced	—	Doubtful	—	—	—	—	—
Deimling et al. (2006)	Coping Orientations to Problems Experienced	—	—	—	—	—	—	Adequate
Fang et al. (2016)	Cardiac Denial of Impact Scale	—	Very good	—	—	—	—	—
Ferrari et al. (2008)	Diagnostic Criteria for Psychosomatic Research	—	Doubtful	—	—	—	—	—
Folks et al. (1988)	Hackett–Cassem Denial Scale	—	—	—	Adequate	—	—	—
Fowers (1992)	Cardiac Denial of Impact Scale	—	—	—	Very good	—	—	—
Fricchione et al. (1992)	Hackett–Cassem Denial Scale	—	Very good	—	—	—	—	—
Froese, Hackett, et al. (1974)	Hackett–Cassem Denial Scale	Doubtful	—	—	—	—	—	—
Froese, Vasquez, et al. (1974)	Hackett–Cassem Denial Scale	—	—	Very good	—	—	—	—
Ganasegeran and Rashid (2017)	Havik and Mæland Denial Scale	—	—	—	—	—	Very good	—
Gattellari et al. (1999)	Cardiac Denial of Impact Scale	—	Adequate	—	—	—	—	—
González‐Freire et al. (2010)	Coping Orientations to Problems Experienced	—	—	—	—	—	—	Adequate
Grandi et al. (2001)	Diagnostic Criteria for Psychosomatic Research	—	Doubtful	—	—	—	—	—
Grassi et al. (1999)	Illness Behaviour Questionnaire	—	Adequate	—	—	—	—	—
Grassi et al. (2005)	Diagnostic Criteria for Psychosomatic Research	—	Doubtful	—	—	—	—	—
Guidi et al. (2013)	Diagnostic Criteria for Psychosomatic Research	—	Doubtful	—	—	—	—	—
Hackett and Cassem (1974)	Hackett–Cassem Denial Scale	Adequate	Adequate	—	—	—	—	—
Hart et al. (2000)	Coping Orientations to Problems Experienced	—	—	—	—	—	—	Adequate
Havik and Mæland 1986	Havik and Mæland Denial Scale	—	—	—	Very good	—	—	—
Huntley et al. (2019)	Cardiac Denial of Impact Scale	—	—	—	—	—	Adequate	—
Ironson et al. (1994)	Coping Orientations to Problems Experienced	—	—	—	—	—	—	Adequate
Jacobsen and Lowery (1992)	Levine Denial of Illness Scale	Doubtful	—	—	Adequate	—	—	—
Kamen et al. (2012)	Coping Orientations to Problems Experienced	—	—	—	—	—	Adequate	—
Karadere et al. (2019)	Acceptance and Action Diabetes Questionnaire	—	—	—	Adequate	—	—	—
Kortte et al. (2007)	Levine Denial of Illness Scale	—	—	—	Very good	—	—	—
Leserman et al. (2000)	Coping Orientations to Problems Experienced	—	—	—	—	—	Adequate	—
Levenson et al. (1984)	Hackett–Cassem Denial Scale	—	Adequate	—	—	—	—	—
Levenson et al. (1989)	Hackett–Cassem Denial Scale	—	Very good	—	—	—	—	—
Levine et al. (1987)	Levine Denial of Illness Scale	Very good	Adequate	—	Very good	—	—	—
Levine et al. (1994)	Levine Denial of Illness Scale	Very good	—	—	—	—	—	—
Mann et al. (2018)	Coping Orientations to Problems Experienced	—	Very good	—	—	—	—	—
McGann et al. (2008)	Levine Denial of Illness Scale	Very good	Very good	—	—	—	—	—
Nazarian et al. (2006)	Coping Orientations to Problems Experienced	—	—	—	—	—	—	Doubtful
Noy et al. (1995)	Hackett–Cassem Denial Scale	—	—	—	Adequate	—	—	—
O'Carroll et al. (2001)	Cardiac Denial of Impact Scale	—	Adequate	—	—	—	—	—
Paredes et al. (2012)	Coping Orientations to Problems Experienced	—	—	—	—	—	Adequate	—
Perkins‐Porras et al. (2008)	Cardiac Denial of Impact Scale	—	—	—	—	—	Adequate	—
Picardi et al. (2005)	Diagnostic Criteria for Psychosomatic Research	—	Doubtful	—	—	—	—	—
Piolanti et al. (2019)	Diagnostic Criteria for Psychosomatic Research	—	Doubtful	—	—	—	—	—
Porcelli et al. (2000)	Diagnostic Criteria for Psychosomatic Research	—	Doubtful	—	—	—	—	—
Pugi et al. (2022)	Illness Denial Questionnaire	—	—	—	—	—	—	Adequate
Rafanelli et al. (2013)	Diagnostic Criteria for Psychosomatic Research	—	Adequate	—	—	—	—	—
Rafanelli et al. (2003)	Diagnostic Criteria for Psychosomatic Research	—	Doubtful	—	—	—	—	—
Ramanathan‐Elion et al. (2016)	Levine Denial of Illness Scale	—	—	—	—	—	Adequate	—
Rossi Ferrario et al. (2017)	Illness Denial Questionnaire	—	Adequate	—	Adequate	—	—	—
Rossi Ferrario et al. (2019)	Illness Denial Questionnaire	—	—	—	Adequate	Very good	—	—
Rossi Ferrario and Panzeri (2020)	Illness Denial Questionnaire	—	Adequate	—	Adequate	—	—	—
Roussi et al. (2007)	Coping Orientations to Problems Experienced	—	Adequate	—	—	—	—	—
Saito et al. (2018)	Acceptance and Action Diabetes Questionnaire	—	—	—	Adequate	—	—	—
Santos et al. (2006)	Denial of Illness Scale	—	Very good	—	—	—	—	—
Schmitt et al. (2014)	Acceptance and Action Diabetes Questionnaire	—	Very good	—	—	—	—	—
Sherman et al. (2000)	Coping Orientations to Problems Experienced	—	Adequate	—	—	—	—	—
Spiess et al. (1995)	Hackett–Cassem Denial Scale	—	Adequate	—	—	—	—	—
Stenström et al. (2005)	Hackett–Cassem Denial Scale	—	Adequate	—	—	—	—	—
Tesio et al. (2019)	Diagnostic Criteria for Psychosomatic Research	—	Doubtful	—	—	—	—	—
Trijsburg et al. (1987)	Denial Questionnaire	—	Very good	—	—	—	—	—
Umucu and Lee (2020)	Coping Orientations to Problems Experienced	—	—	—	—	—	—	Adequate
Vos et al. (2007)	Denial of Cancer Interview	Very good	—	—	—	—	—	—
Vos et al. (2008)	Denial of Cancer Interview	—	Very good	—	—	—	—	—
Vos et al. (2010)	Denial of Cancer Interview	—	Adequate	—	—	—	—	—
Vos et al. (2011)	Denial of Cancer Interview	—	Adequate	—	—	—	—	—
Warrenburg et al. (1989)	Levine Denial of Illness Scale	—	—	—	Adequate	—	—	—
Weaver et al. (2004)	Coping Orientations to Problems Experienced	—	—	—	—	—	Adequate	—
White et al. (2016)	Cardiac Denial of Impact Scale	—	—	—	—	—	Adequate	—
Wijk et al. (2024)	Acceptance and Action Diabetes Questionnaire	—	Adequate	—	Very good	Very good	—	—
Yellowlees and Ruffin (1989)	Illness Behaviour Questionnaire	—	Doubtful	—	—	—	—	—

**TABLE 4:** Levels of evidence for the overall quality of clinimetric properties of each instrument of illness denial.

Instruments	Clinimetric properties	Levels of evidence
**Patient‐reported outcome measures (PROMs)**		
Acceptance and Action Diabetes Questionnaire	Clinimetric sensitivity Concurrent validity Construct validity	Strong Strong Strong
Cardiac Denial of Impact Scale	Clinimetric sensitivity Concurrent validity Incremental validity	Strong Strong Strong
Coping Orientations to Problems Experienced	Clinimetric sensitivity Incremental validity Predictive validity	Strong Strong Strong
Denial Questionnaire	Clinimetric sensitivity	Strong
Havik and Mæland Denial Scale	Concurrent validity Incremental validity	Strong Strong
Illness Behaviour Questionnaire	Clinimetric sensitivity	Strong
Illness Denial Questionnaire	Clinimetric sensitivity Concurrent validity Construct validity Predictive validity	Strong Strong Strong Moderate
Clinician‐rated instruments		
Denial of Illness Scale	Clinimetric sensitivity	Strong
Diagnostic Criteria for Psychosomatic Research	Clinimetric sensitivity	Moderate
Hackett–Cassem Denial Scale	Inter‐rater reliability Clinimetric sensitivity Clinical validity Concurrent validity	Moderate Strong Strong Strong
Levine Denial of Illness Scale	Inter‐rater reliability Clinimetric sensitivity Concurrent validity Incremental validity	Strong Strong Strong Moderate
Denial of Cancer Interview	Inter‐rater reliability Clinimetric sensitivity	Strong Strong

### 3.1 Acceptance and Action Diabetes Questionnaire (AADQ)

#### 3.1.1 Clinimetric Sensitivity

The short version of the Acceptance and Action Diabetes Questionnaire (AADQ), which only included six items, sensitively differentiated those with low levels of illness denial from patients with higher illness denial who reported lower treatment satisfaction, higher diabetes distress, more depressive symptoms, lower health‐related quality of life and worse glycaemic control (Schmitt et al. 2014). In subsequent research (Wijk et al. 2024), a Person Separation Reliability Index (PSI) of 0.81 was found for a brief version of the AADQ, which only consisted of nine items (AADQ_9_). The PSI value of 0.81 indicated that the AADQ_9_ could reliably distinguish between groups of patients with different levels of illness denial (Wijk et al. 2024).

#### 3.1.2 Concurrent Validity

In the validation study of the Japanese version of the AADQ, Saito et al. (2018) showed that the eight‐item version of the instrument significantly and negatively correlated with glycaemic control. Wijk et al. (2024) also found a statistically significant and negative correlation between the AADQ_9_ and measures of depression, anxiety and stress. Similar results were reported by Karadere et al. (2019) who found statistically significant correlations between the Turkish version of the AADQ and measures of anxiety, depression and psychological distress.

#### 3.1.3 Construct Validity

The AADQ_9_ was found to achieve an overall fit to the Rasch model (Wijk et al. 2024). Dimensionality analyses yielded less than 5% of significant *t*‐tests, thus indicating that AADQ_9_ was a unidimensional instrument of illness denial (Wijk et al. 2024).

### 3.2 Cardiac Denial of Impact Scale (CDIS)

#### 3.2.1 Clinimetric Sensitivity

Gattellari et al. (1999) developed a modified version of the Cardiac Denial of Impact Scale (CDIS) for evaluating the extent to which patients minimize the emotional impact of having cancer and deny feeling worried or afraid. They showed that the instrument sensitively distinguished between patients with high levels of illness denial and those with relatively low denial (Gattellari et al. 1999). In a subsequent study, the original version of the CDIS sensitively discriminated between those who believed that they were having a heart attack and patients waiting more than 4 h prior to seeking medical help who had significantly higher levels of illness denial (O'Carroll et al. 2001). In a cross‐sectional study, the CDIS sensitively differentiated those with low illness denial from patients with a high level of denial who were less likely to suffer from depression, anxiety and suboptimal well‐being in the 6 months prior to the onset of a ST‐segment elevation myocardial infarction (Fang et al. 2016). Symptomatic differences between patients with low denial and those with higher levels of illness denial were also detected (Fang et al. 2016). The CDIS revealed that cardiac patients with higher illness denial tended to perceive lower pain and less racing heart and were less likely to recognize symptoms of myocardial infarction (Fang et al. 2016).

#### 3.2.2 Concurrent Validity

A statistically significant and negative correlation between the CDIS and the Psychological Distress Scale and the Physical Symptom Checklist was found, thus supporting the concurrent validity of the CDIS (Fowers 1992).

#### 3.2.3 Incremental Validity

The tendency to deny emotional distress associated with heart disease was found to increase the prediction of depression, thus supporting the incremental validity of the CDIS (Huntley et al. 2019). In another study, Perkins‐Porras et al. (2008) showed that each one‐unit increase in levels of cardiac denial significantly increased the likelihood of longer pre‐hospital delay, thus providing further support for the incremental validity of the CDIS. The CDIS was also found to increase the prediction of non‐adherence to cardiac care (White et al. 2016).

### 3.3 COPE

#### 3.3.1 Clinimetric Sensitivity

The Coping Orientations to Problems Experienced (COPE) denial subscale was found to be highly sensitive to changes in levels of denial over time (Carver et al. 1993). The subscale revealed that denial decreased significantly from pre‐surgery to post‐surgery with a further, more gradual reduction from post‐surgery to the 1‐year follow‐up (Carver et al. 1993). Roussi et al. (2007) reported a similar trend showing that the COPE denial subscale sensitively detected changes in levels of denial over time. In another study, the COPE denial subscale sensitively distinguished patients in the pre‐treatment group from individuals receiving active treatment or in the immediate aftermath of treatment who reported greater use of illness denial (Sherman et al. 2000). The COPE denial subscale was also found to sensitively differentiate patients with chronic pain without neuropathic features from those with chronic pain with neuropathic characteristics who were more likely to use illness denial as a coping strategy (Mann et al. 2018). In a subsequent study, the COPE denial subscale sensitively discriminated between patients with psoriasis and healthy controls who used illness denial significantly more (Cvitanović et al. 2020).

#### 3.3.2 Incremental Validity

The COPE denial subscale added a unique contribution in the prediction of postoperative psychological distress (Carver et al. 1993). In another research, it was found that the risk of AIDS was doubled for every one‐unit increase in levels of denial, thus supporting the incremental validity of the COPE denial subscale (Leserman et al. 2000). Weaver et al. (2004) showed that illness denial as evaluated with the COPE denial subscale was a significant predictor of poor quality of life, over and beyond the effects of sociodemographic and disease‐related variables. Kamen et al. (2012) reported similar results showing that levels of quality of life remained low in HIV patients who used illness denial as a coping strategy. Paredes et al. (2012) found that the COPE denial subscale contributed to a significant increase in the amount of explained variance for the prediction of depression.

#### 3.3.3 Predictive Validity

Scores of the COPE illness denial subscale were found to be significant predictors of disease progression to AIDS (Ironson et al. 1994). In another study, illness denial was a significant predictor of greater pain severity (Hart et al. 2000). Nazarian et al. (2006) reported similar findings showing that the COPE subscale of illness denial was a significant predictor of somatic symptoms. The COPE subscale on illness denial was also found to significantly predict symptoms of anxiety and depression in patients with long‐term cancer (Deimling et al. 2006). The COPE denial subscale was also found to be a significant predictor of poor prognosis in patients with higher levels of illness denial who were more likely to end up in the hospital for acute exacerbations of their asthma (González‐Freire et al. 2010). In a more recent study, the COPE illness denial subscale was found to be a significant predictor of well‐being in patients with self‐reported chronic conditions and disabilities (Umucu and Lee 2020).

### 3.4 Denial of Illness Scale (DIS)

#### 3.4.1 Clinimetric Sensitivity

Santos et al. (2006) found that the Denial of Illness Scale (DIS) sensitively differentiated individuals with acute stroke from acute coronary patients. They showed that, compared to those with acute coronary disease, stroke patients denied more frequently and intensely their fear of death (Santos et al. 2006).

### 3.5 Denial Questionnaire (DQ)

#### 3.5.1 Clinimetric Sensitivity

The Denial Questionnaire (DQ) was found to sensitively discriminate between partners and patients who, when looking back on the pre–acute myocardial infarction period, had the tendency to see themselves as having been less resentful, dependent, anxious and exhausted than their significant others judged them to have been (Trijsburg et al. 1987). In a subsequent follow‐up study, the DQ sensitively differentiated individuals waiting longer than half an hour prior to seeking medical help from acute myocardial infarction patients who asked for medical help within half an hour and had the tendency to deny their feelings of resentment and vital exhaustion to a lesser degree (Bleeker et al. 1995).

### 3.6 Diagnostic Criteria for Psychosomatic Research (DCPR)

#### 3.6.1 Clinimetric Sensitivity

With the use of the semi‐structured interview for Diagnostic Criteria for Psychosomatic Research (DCPR), illness denial was detected in 3.7% of patients with functional gastrointestinal disorders (Porcelli et al. 2000). In heart transplant patients, illness denial was documented in 4.6% of the sample (Grandi et al. 2001). DCPR sensitively revealed reactions of illness denial in 3.3% of patients with first myocardial infarction (Rafanelli et al. 2003). In patients with various dermatological disorders, illness denial was found in 1.8% of the sample (Picardi et al. 2005). Illness denial was also sensitively detected in 8.2% of patients with different forms of cancer (Grassi et al. 2005). In another study with primary care patients, the DCPR were found to be highly sensitive to reactions of illness denial, which were detected in 68% of the sample (Ferrari et al. 2008). DCPR sensitively detected illness denial in 22.9% of patients with congestive heart failure (Guidi et al. 2013) and in 23.9% of patients with suspected vasovagal syncope (Rafanelli et al. 2013). Illness denial was also detected in 13.4% of kidney transplant patients (Battaglia et al. 2018). DCPR were found to be highly sensitive to reactions of illness denial, which were detected in 32.7% of patients with fibromyalgia and in 20.4% of those with rheumatoid arthritis (Tesio et al. 2019). In another study with primary care patients, illness denial was documented in 3.5% of the sample (Piolanti et al. 2019).

### 3.7 Hackett–Cassem Denial Scale (HCDS)

#### 3.7.1 Inter‐Rater Reliability

Reliability coefficients between two independent raters for two series of 10 patients were 0.84 and 0.89 respectively, indicating an excellent level of inter‐clinician agreement (Hackett and Cassem 1974). Froese, Hackett, et al. (1974) tested the measurement properties of a brief version of the Hackett–Cassem Denial Scale (HCDS) that only included 12 items, reporting an inter‐rater reliability coefficient of 0.78, which indicates a good level of inter‐rater agreement.

#### 3.7.2 Clinimetric Sensitivity

Using a one‐way analysis of variance, Hackett and Cassem (1974) showed that the HCDS sensitively discriminated between mild denial (i.e., a term used to describe patients who either complained of anxiety symptoms or who readily admitted being frightened) and severe denial (i.e., those who stated unequivocally that they felt no fear at any time throughout their hospital stay or earlier in their lives). The HCDS was also found to sensitively differentiate partial denial (i.e., patients who initially denied being frightened, but eventually admitted feeling at least some fear) from those with severe denial (Hackett and Cassem 1974). In a subsequent study, the HCDS sensitively distinguished deniers from non‐deniers (Levenson et al. 1984). The HCDS revealed that patients with illness denial reached medical stabilization much more rapidly than those without illness denial (Levenson et al. 1984). The same research group reported similar findings in another study, where the HDCS sensitively discriminated between unstable angina pectoris patients with mild illness denial and those with major denial who were more likely to reach medical stabilization (Levenson et al. 1989). Fricchione et al. (1992) proposed a modified version of the HCDS that included 19 items only. They found that this short version of the HCDS sensitively differentiated patients with high levels of illness denial from those with minimal denial who displayed more interpersonal sensitivity, had more anxiety and depression and reported greater sleep disturbances (Fricchione et al. 1992). In a clinical trial, the HCDS was sensitive to changes in levels of illness denial, which significantly decreased 3 and 9 months after the end of the intervention (Spiess et al. 1995). The HCDS was also found to sensitively differentiate non‐delayers from myocardial infarction patients with higher levels of illness denial who showed a prolonged delay in going to the hospital (Stenström et al. 2005).

#### 3.7.3 Clinical Validity

In a validation study, where a 36‐item version of the HCDS was used, one rater evaluated the intensity of illness denial on the basis of clinical judgement, whereas the other assessed each patient using the HCDS (Froese, Vasquez, et al. 1974). A statistically significant and positive association between the severity of illness denial as assessed with the HCDS and the clinical judgement was found, thus supporting the clinical validity of the HCDS (Froese, Vasquez, et al. 1974).

#### 3.7.4 Concurrent Validity

In a prospective study, where a modified version of the HCDS was used, a statistically significant and negative correlation between the HCDS and measures of anxiety, depression and psychosocial adjustment was found (Folks et al. 1988). Using the original version of the HCDS, Noy et al. (1995) revealed a statistically significant and negative correlation between illness denial and depression.

### 3.8 Havik and Mæland Denial Scale (HMDS)

#### 3.8.1 Concurrent Validity

Statistically significant and positive correlations between the Havik and Mæland Denial Scale (HMDS) and the Myocardial Infarction Attitude Scale, the Beck Hopelessness Scale and the Attitude Towards the Hospital Staff Scale were found, thus indicating that those with higher illness denial showed more optimistic attitudes, lower levels of hopelessness and greater satisfaction with the hospital staff (Havik and Mæland 1986).

#### 3.8.2 Incremental Validity

A one‐unit increase in the denial of illness was found to be associated with a 20% increase in the odds of being non‐adherent to medications, thus supporting the incremental validity of the HMDS (Ganasegeran and Rashid 2017).

### 3.9 Illness Behaviour Questionnaire (IBQ)

#### 3.9.1 Clinimetric Sensitivity

Yellowlees and Ruffin (1989) found that patients with asthma significantly increased their levels of illness denial after a life‐threatening attack of asthma, thus showing that the Illness Behaviour Questionnaire (IBQ) was highly sensitive to changes in levels of denial over time. In another study, the IBQ denial subscale was found to sensitively differentiate non‐cases from cases with a near fatal attack of asthma who reported significantly higher levels of denial (Campbell et al. 1995). The denial subscale was also found to sensitively discriminate between individuals with HIV and cancer patients who scored significantly higher on an expanded version of the IBQ (Grassi et al. 1999). In a prospective, randomized controlled trial, the original version of the IBQ sensitively distinguished those not having a history of emergency visits from patients with history of emergency visits who had significantly higher levels of denial (Adams et al. 2001). In a subsequent study, the IBQ subscale on denial sensitively differentiated individuals without a repressive coping style from patients with a repressive coping style (i.e., those with low trait anxiety and high defensiveness) who exhibited significantly higher levels of illness denial (Cooke et al. 2003).

### 3.10 Illness Denial Questionnaire (IDQ)

#### 3.10.1 Clinimetric Sensitivity

In the validation study of the Illness Denial Questionnaire (IDQ), analysis of variance revealed significant differences between the group of patients with renal diseases and both the oncological and respiratory groups (Rossi Ferrario et al. 2017). The IDQ was also found to sensitively discriminate between cardiac and respiratory patients (Rossi Ferrario et al. 2017). In another study, the IDQ was found to be highly sensitive to changes in levels of illness denial over time (Rossi Ferrario and Panzeri 2020). At discharge from cardiac rehabilitation, a significant worsening of illness denial was detected on the IDQ subscales of denial of negative emotions and resistance to change (Rossi Ferrario and Panzeri 2020).

#### 3.10.2 Concurrent Validity

The IDQ was found to be significantly and negatively correlated with measures of anxiety and depression (Rossi Ferrario et al. 2017). A negative and moderate correlation between the eight‐item version of the IDQ and measures of anxiety and depression was also found (Rossi Ferrario et al. 2019). In another study, Rossi Ferrario and Panzeri (2020) reported similar findings showing a statistically significant and negative association between the original 24‐item version of the IDQ and the short form of the Anxiety and Depression Questionnaire.

#### 3.10.3 Construct Validity

The eight‐item version of the IDQ was found to fit the Rasch model expectations of unidimensionality (Rossi Ferrario et al. 2019).

#### 3.10.4 Predictive Validity

Scores on the IDQ denial of negative emotions subscale were significant predictors of higher health‐related quality of life in patients with chronic kidney disease (Pugi et al. 2022).

### 3.11 Levine Denial of Illness Scale (LDIS)

#### 3.11.1 Inter‐Rater Reliability

Levine et al. (1987) found an intra‐class correlation coefficient for the Levine Denial of Illness Scale (LDIS) total score of 0.78, which indicated an excellent level of inter‐rater agreement. In a subsequent study, Jacobsen and Lowery (1992) showed that two independent interviewers agreed 75% of the time in their scoring of the LDIS items, thus reporting an acceptable level of inter‐rater reliability. In a study involving patients with various medical disorders, the average intra‐class correlation coefficient for the LDIS items measuring cognitive denial was 0.66, thus suggesting a good consistency between raters (Levine et al. 1994). The average intra‐class correlation coefficient for the remaining LDIS items assessing affective denial was 0.61, which indicated a good level of inter‐rater agreement (Levine et al. 1994). McGann et al. (2008) reported similar results. Cohen's kappa coefficients for the LDIS subscales of cognitive and affective illness denial were 0.81 and 0.96, respectively, both denoting an overall good level of agreement between raters (McGann et al. 2008).

#### 3.11.2 Clinimetric Sensitivity

The LDIS subscale of cognitive denial was found to sensitively discriminate between patients with coronary artery disease and those who had a diagnosis of epilepsy, hypertension or stroke (Levine et al. 1994). Coronary artery disease patients scored significantly higher on the LDIS subscale of cognitive denial than the other ones (Levine et al. 1994). In a subsequent study, McGann et al. (2008) showed that the LDIS subscales of cognitive and affective denial sensitively differentiated asthma patients with suboptimal compliance from those with optimal compliance. They found that asthma patients with suboptimal compliance had significantly higher information avoidance and affective denial than patients with optimal compliance (McGann et al. 2008).

#### 3.11.3 Concurrent Validity

A statistically significant and negative correlation between the LDIS and the Symptom Checklist‐90‐Revised (SCL‐90‐R) subscales of anxiety and depression was found (Levine et al. 1987). There was also a statistically significant and negative correlation between the LDIS and the SCL‐90‐R total score, indicating that, compared with low deniers, individuals with high levels of illness denial are generally less distressed (Levine et al. 1987). The authors also showed that the LDIS was inversely correlated with the number of days in the intensive care unit and with the presence of atrial arrhythmias, indicating that high deniers spent fewer days in intensive care and had fewer signs of cardiac dysfunction than patients with low levels of illness denial (Levine et al. 1987). In a subsequent study with patients who underwent a stress interview while their blood pressure was evaluated, Warrenburg et al. (1989) found that LDIS was associated not only with lower systolic blood pressure reactivity but also with lower cumulative elevation in systolic and diastolic blood pressure over the course of the interview. Jacobsen and Lowery (1992) reported similar findings, showing that LDIS was significantly and negatively correlated with both measures of anxiety and depression. In another study, Kortte et al. (2007) found that there was a negative correlation between the Hopkins Rehabilitation Engagement Rating Scale and the LDIS, thus indicating that lower rehabilitation engagement was significantly related to higher levels of illness denial.

#### 3.11.4 Incremental Validity

In a prospective cohort study, LDIS accounted for a significant increase in the prediction of engagement in the rehabilitation process (Ramanathan‐Elion et al. 2016). Greater illness denial significantly increased the prediction of lower levels of rehabilitation engagement (Ramanathan‐Elion et al. 2016).

### 3.12 The Denial of Cancer Interview (DCI)

#### 3.12.1 Inter‐Rater Reliability

Cohen's kappa coefficients indicated a satisfactory level of agreement between two raters who independently scored the first part of the Denial of Cancer Interview (DCI) (Vos et al. 2007).

#### 3.12.2 Clinimetric Sensitivity

The DCI was found to be highly sensitive to changes in levels of illness denial over time (Vos et al. 2008). The DCI revealed that the mean level of illness denial was low at baseline and significantly increased thereafter (Vos et al. 2008). The DCI was also found to sensitively differentiate lung cancer patients with low illness denial from cancer patients showing moderate to severe levels of denial who reported better physical functioning, less nausea and vomiting and less appetite loss and dyspnoea (Vos et al. 2010). In a subsequent longitudinal study, Vos et al. (2011) showed that the DCI sensitively discriminated between those with low denial and patients with moderate levels of illness denial who reported better quality of life and emotional functioning, less anxiety and depression.

## 4 Discussion

The findings of this critical review indicate that the existing instruments of illness denial entail different clinimetric properties, which may guide the choice of the most suitable measure based on a number of clinical factors (e.g., illness severity and its rate of progression) that have for a long time been neglected in customary taxonomy (Feinstein et al. 1986). This implies that there is not a unique measure of illness denial, which applies to all situations, but clinical investigators and practitioners are asked to determine the purpose of the assessment process and select the instruments that best fit with the clinical setting and the specific clinical population for which the evaluation tools will be used. Accordingly, as Feinstein et al. (1986) noted, if the main purpose and setting of the evaluation process have not been clearly defined, a measure is unlikely to be of any clinical utility no matter how high its previous statistical coefficients may have been. The available instruments of illness denial that have been analysed in the present work can therefore be used in certain clinical settings and for specific clinical purposes and, most importantly, selected according to their clinical utility.

### 4.1 Screening Utility

The term screening refers to the ability of a measure to identify a clinical condition or syndrome that is asymptomatic at the time of performing the test (Bech 2012; Sherman 2011). Thus, screening measures are conventionally used as first‐line evaluation methods not only to early detect individuals at increased risk for clinical complications but also to make critical decisions about the need for further evaluations (Bufka and Camp 2010; Robins 2020).

The AADQ (Gregg et al. 2007) was found to possess screening utility and it can be used for early detection of symptoms of psychological distress in diabetes patients presenting with illness denial (Karadere et al. 2019; Schmitt et al. 2014; Wijk et al. 2024). The DIS (Starkstein et al. 1993) is another instrument that may have considerable screening utility. Given the brevity and simplicity of this easy‐to‐use index, the DIS can be used as a screening tool of illness denial in stroke patients. Future studies are, however, needed to further assess its screening potential.

### 4.2 Diagnostic Utility

Diagnostic utility refers to the use of instruments that may help clinicians rule in or rule out the presence of a condition (Bech 2012; Carrozzino et al. 2021).

The DCPR can be used from a diagnostic point of view to improve the detection of illness denial. The semi‐structured interview for DCPR indeed proved to be sensitive to the presence of illness denial across a wide range of clinical conditions; thus, it has the potential to be used as a transdiagnostic measure (Battaglia et al. 2018; Ferrari et al. 2008; Grandi et al. 2001; Grassi et al. 2005; Guidi et al. 2013; Picardi et al. 2005; Piolanti et al. 2019; Porcelli et al. 2000; Rafanelli et al. 2013; Rafanelli et al. 2003; Tesio et al. 2019). However, it should be noted that the criteria and items of the semi‐structured interview for DCPR have been designed to identify the negative outcomes (e.g., delay in seeking medical help and reduced treatment compliance) associated with the maladaptive manifestations of illness denial; thus, their use may be of limited clinical utility in particular clinical situations, where illness denial was found to have an important protective value (Patierno et al. 2023). This is the main reason why DCPR should be supplemented by another semi‐structured interview such as the LDIS (Levine et al. 1987), which might be used to identify the complex manifestations of illness denial. The LDIS (Levine et al. 1987) has been shown to be a reliable and valid clinical interview (Jacobsen and Lowery 1992; Levine et al. 1994; McGann et al. 2008), and it can be used as a transdiagnostic measure to recognize the affective, behavioural and cognitive components of illness denial.

The use of assessment instruments with optimal inter‐rater reliability appears to be highly relevant, particularly in contexts where diagnostic procedures are employed. Wright and Feinstein (1992) clarified the clinical significance of inter‐rater reliability noting that regardless of how sensible, easy and useful an instrument may seem to be, it is ultimately unsuccessful if its process cannot be replicated or its results reproduced. The measurement property of inter‐rater reliability is not intended to replace clinical judgement given that the raters' clinical experience and their level of training and expertise in the use of assessment instruments play a significant role in eliciting the necessary information during the interview process (Carrozzino et al. 2020). As Bech (2012) consistently observed, in the clinimetric approach, the experienced clinician must be the key, and subsequent analysis is made of the percentage of clinical investigators using a certain instrument that deviate from the master. This implies that the effective use of highly reliable instruments such as the LDIS depends entirely on the clinical skills and experience of the interviewer.

### 4.3 Staging Utility

Staging utility refers to the ability of an instrument to assess the severity of a clinical condition and also includes the evaluation of the rate of growth or progression of the disease (Feinstein 1982).

The AADQ (Gregg et al. 2007), the HCDS (Hackett and Cassem 1974), the IDQ (Rossi Ferrario et al. 2017) and the DCI (Vos et al. 2007) have been found to be appropriate instruments of staging. The AADQ, particularly the AADQ_9_ (Wijk et al. 2024), is recommended for assessing the degree of illness denial in patients with diabetes, whereas the IDQ may be better suited for the evaluation of severity and rate of progression of illness denial in patients with various clinical conditions (Rossi Ferrario and Panzeri 2020; Rossi Ferrario et al. 2019). Given that the DCI (Vos et al. 2007) has been shown to sensitively detect changes in levels of illness denial over time (Vos et al. 2008; Vos et al. 2010; Vos et al. 2011), it can be used for longitudinal monitoring of the severity and impact of illness denial in patients with various forms of lung cancer.

Regarding the HCDS, it was found to be a highly reliable and valid instrument that can be used to assess the degree of illness denial in patients with several cardiac diseases (Froese, Hackett, et al. 1974; Hackett and Cassem 1974; Levenson et al. 1989; Stenström et al. 2005). The assessment of the severity of illness denial is crucial to understand its impact on health attitudes and behaviours. In their systematic review, Patierno et al. (2023) clearly showed that, compared to patients with higher levels of illness denial, those with mild illness denial did not delay in reporting their symptoms to the medical attention, were more likely to display health‐promoting attitudes and behaviour and had more positive clinical outcomes (Fang et al. 2016; Fricchione et al. 1992; Hackett and Cassem 1969; Lehto et al. 2006; Levenson et al. 1989; Levine et al. 1987; Pugi et al. 2022; Vos et al. 2010). The clinical distinction between excessive and optimal levels of illness denial thus requires a grading process, an evaluation procedure that is consistent with the clinimetric approach, where criteria for detecting the presence of clinical entities should be supplemented by criteria of gradation for the assessment of symptom severity (Feinstein 1982).

Grading the intensity of illness denial is, however, only the initial step of the evaluation process according to the clinimetric method of staging (Fava 2024), which involves the assessment of the longitudinal course of illness denial. The use of highly sensitive and valid indices such as the AADQ (Gregg et al. 2007), the HCDS (Hackett and Cassem 1974), the IDQ (Rossi Ferrario et al. 2017) and the DCI (Vos et al. 2007) should therefore be supplemented by the clinimetric method of staging to identify the prodromal, acute and residual phases of illness denial and understand its progression over time. Freyberger et al. (1979) were the first to propose a staging model of illness denial, which has been originally applied to renal transplant patients. This staging model (Freyberger et al. 1979) can be used to differentiate the following three levels of severity of illness denial:

1. The realistically adapted denial, which consists of the ability to perceive and sufficiently control the disease situation. The realistically adapted denial usually occurs in the majority of cases and is adequately tolerated by the patient, who is very cooperative and able to accept the therapeutic measures.
2. The highly intensive denial, which refers to a nearly total repudiation or suppression of the disease situation and of the inherent feelings, as well as of the understanding concerning the therapeutic necessity.
3. The insufficient denial, in which the patient has the tendency to overestimate the disease situation and is more likely to experience hopelessness and/or symptoms of anxiety. The clinical utility and the potential transdiagnostic applicability of this staging model (Freyberger et al. 1979) to patients with different medical conditions need to be evaluated in future studies.

### 4.4 Prognostic Utility

Prognostic utility can be defined as the degree to which a certain instrument is able to predict outcomes that may demarcate major therapeutic and prognostic differences among patients who otherwise seem to be deceptively similar because they share the same medical diagnosis (Wright and Feinstein 1992).

The CDIS (Fowers 1992), the COPE denial subscale (Carver et al. 1989), the DQ (Trijsburg et al. 1987), the IBQ (Pilowsky and Spence 1975) and the HMDS (Havik and Mæland 1986) appear to be instruments that may provide important prognostic information. The CDIS and DQ were found to predict the tendency of some cardiac patients with illness denial to delay seeking medical advice (Bleeker et al. 1995; Fang et al. 2016; O'Carroll et al. 2001; Perkins‐Porras et al. 2008), whereas the HMDS improved the prediction of poor treatment adherence in post‐myocardial infarction patients with illness denial (Ganasegeran and Rashid 2017). The use of CDIS, DQ and HMDS in patients with cardiac disorders, where the early recognition of life‐threatening diseases such as myocardial infarction is crucial to reduce the risk of mortality, may therefore have major prognostic implications. The same recommendations apply to the IBQ (Pilowsky and Spence 1975), which was found to have considerable prognostic utility, particularly in patients with a diagnosis of asthma, where high levels of illness denial may affect the patients' ability to recognize and promptly respond to life‐threatening attacks of asthma (Campbell et al. 1995; Yellowlees and Ruffin 1989). Regarding the COPE denial subscale (Carver et al. 1989), it can be used to prognosticate a future state, including disease progression, particularly in HIV patients, where illness denial was a significant predictor of poor clinical outcomes (Hart et al. 2000; Ironson et al. 1994; Leserman et al. 2000; Weaver et al. 2004).

### 4.5 Strengths and Limitations

The major conclusions of this critical review are supported not only by the high methodological quality of included studies but also by the strong levels of evidence, which were found, in particular, for the clinimetric sensitivity of PROMs and clinician‐rated instruments of illness denial. However, certain findings should be interpreted in light of some limitations. First, the current literature still lacks an in‐depth analysis of the staging utility of the available measures of illness denial: few investigators have attempted to study illness denial longitudinally and the staging utility of existing instruments was evaluated in only a limited number of studies using clinimetric criteria of dimensionality and scalability (Bech 2012). Construct validity of the AADQ_9_ was assessed using Rasch analysis but a cross‐sectional design was applied to collect data (Wijk et al. 2024). Rasch analysis was also used to evaluate the construct validity of IDQ (Rossi Ferrario et al. 2019), but only one study adopted a longitudinal design to assess the staging utility of this instrument (Rossi Ferrario and Panzeri 2020). The staging utility of DCI was assessed in several longitudinal studies (Vos et al. 2007, 2008, 2010, 2011) but validation studies employing Rasch and Mokken analyses are remarkably lacking. The same problem was observed with the HCDS (Hackett and Cassem 1974), which was never analysed using Rasch and Mokken analyses. This implies that future longitudinal studies, in which Rasch and Mokken (Bech 2012) analyses are employed, are needed to further assess whether the AADQ (Gregg et al. 2007), the HCDS (Hackett and Cassem 1974), the IDQ (Rossi Ferrario et al. 2017) and the DCI (Vos et al. 2007) represent valid instruments of illness denial severity in patients with different medical disorders. Second, in most of the retrieved studies, little or no information is provided regarding the patient–clinician relationship, which may significantly affect the process of assessment of illness denial (Patierno et al. 2023). Lack of adequate information or misunderstandings that may occur during medical encounters may influence the performance of existing instruments of illness denial, thus introducing a potential source of bias that should be carefully considered in future studies.

## 5 Conclusions

Existing instruments of illness denial should not be viewed as conflicting or mutually exclusive evaluation methods but, rather, as complementary tools that can be used jointly for a comprehensive assessment of illness denial in patients with medical disorders. Their routine use in clinical research and daily practice is not intended to replace clinical judgement and reasoning but should increasingly support and inform clinical decisions to foster meaningful connections between clinicians and patients and tailor individualized treatment strategies.

The effective use of instruments of illness denial requires careful clinical interviewing with opportunity for discussion and clarification to let patients feel engaged during the entire evaluation process. Indeed, if appropriately questioned about their illness denial, patients may feel understood and validated in their experiences and also provide clinicians with vivid descriptions of their unique ways of perceiving, evaluating and responding to illness. The clinimetric use of instruments of illness denial has considerable prognostic value and the great potential to help patients with medical disorders to early recognize and promptly respond to their illness.

## Funding

The authors have nothing to report.

## Conflicts of Interest

The authors declare no conflicts of interest.

## Supporting information


**Appendix S1:** Supporting Information.

## Data Availability

Data sharing is not applicable to this article as no datasets were generated or analysed during the current study.
