# Supplementary material for: Psychological Assessment of Illness Denial in Medical Settings: A Critical Review
Source: Clin Psychol Psychother. 2026 Feb 16;33(1):e70240. doi: 10.1002/cpp.70240 (PMC12910258; doi:10.1002/cpp.70240)
Supplement: Supplementary file 1 — Appendix S1: Supporting Information. [file CPP-33-e70240-s001.docx]

**Table A.1.** Studies included in the critical review

| **Reference** | **Title** | **Clinical Population** | **Measure of Illness Denial** |
| --- | --- | --- | --- |
| Adams et al., 2001 | A randomized trial of peak‐flow and symptom‐based action plans in adults with moderate‐to‐severe asthma | 134 patients with moderate-to-severe asthma | Illness Behavior Questionnaire |
| Battaglia et al., 2018 | Abnormal illness behavior, alexithymia, demoralization, and other clinically relevant psychosocial syndromes in kidney transplant recipients: a comparative study of the diagnostic criteria for psychosomatic research system versus ICD-10 psychiatric nosology | 134 kidney transplant recipients | Diagnostic Criteria for Psychosomatic Research |
| Bleeker et al., 1995 | Psychological and knowledge factors related to delay of help-seeking by patients with acute myocardial infarction | 300 patients with acute myocardial infarction | Denial Questionnaire |
| Campbell et al., 1995 | Psychiatric and medical features of near fatal asthma | 77 consecutive cases of near fatal asthma attacks | Illness Behavior Questionnaire |
| Carver et al., 1993 | How coping mediates the effect of optimism on distress: a study of women with early stage breast cancer | 59 breast cancer patients | Coping Orientations to Problems Experienced |
| Cooke et al., 2003 | Lung function, adherence and denial in asthma patients who exhibit a repressive coping style | 42 patients with asthma | Illness Behavior Questionnaire |
| Cvitanović et al., 2020 | How to cope with psoriasis: data from patient tests and surveys | 56 patients with psoriasis | Coping Orientations to  Problems Experienced |
| Deimling et al., 2006 | Coping among older‐adult, long‐term cancer survivors | 321 long-term cancer survivors | Coping Orientations to Problems Experienced |
| Fang et al., 2016 | Is denial a maladaptive coping mechanism which prolongs pre-hospital delay in patients with ST-segment elevation myocardial infarction? | 533 patients with diagnosis of ST-elevated myocardial infarction | Cardiac Denial of Impact Scale |
| Ferrari et al., 2008 | Frequent attenders in primary care: impact of medical, psychiatric and psychosomatic diagnoses | 100 primary care patients | Diagnostic Criteria for Psychosomatic Research |
| Folks et al., 1988 | Denial: predictor of outcome following coronary bypass surgery | 121 coronary artery bypass surgery patients | Hackett-Cassem Denial Scale |
| Fowers, 1992 | The Cardiac Denial of Impact Scale: a brief, self-report research measure | 91 cardiac rehabilitation patients | Cardiac Denial of Impact Scale |
| Fricchione et al., 1992 | Psychological adjustment to end-stage renal disease and the implications of denial | 63 end-stage renal disease patients | Hackett-Cassem Denial Scale |
| Froese et al., 1974a | Trajectories of anxiety and depression in denying and nondenying acute myocardial infarction patients during hospitalization | 36 acute myocardial infraction patients | Hackett-Cassem Denial Scale |
| Froese et al., 1974b | Validation of anxiety depression and denial scales in a coronary care unit | 65 acute coronary patients | Hackett-Cassem Denial Scale |
| Ganasegeran and Rashid, 2017 | The prevalence of medication nonadherence in post-myocardial infarction survivors and its perceived barriers and psychological correlates: a cross-sectional study in a cardiac health facility in Malaysia | 242 post-myocardial infarction patients | Havik and Mæland Denial Scale |
| Gattellari et al., 1999 | Misunderstanding in cancer patients: why shoot the messenger? | 244 outpatients with various forms of cancer | Cardiac Denial of Impact Scale |
| González-Freire et al., 2010 | Repression and coping styles in asthmatic patients | 75 asthmatic patients | Coping Orientations to Problems Experienced |
| Grandi et al., 2001 | Psychological evaluation after cardiac transplantation: the integration of different criteria | 129 consecutive patients who underwent heart transplant surgery | Diagnostic Criteria for Psychosomatic Research |
| Grassi et al., 1999 | Illness behavior, emotional stress and psychosocial factors among asymptomatic HIV-infected patients | 73 asymptomatic HIV outpatients | Illness Behavior Questionnaire |
| Grassi et al., 2005 | Use of the diagnostic criteria for psychosomatic research in oncology | 146 cancer patients | Diagnostic Criteria for Psychosomatic Research |
| Guidi et al., 2013 | Assessing psychological factors affecting medical conditions: comparison between different proposals | 70 outpatients with congestive heart failure | Diagnostic Criteria for Psychosomatic Research |
| Hackett and Cassem, 1974 | Development of a quantitative rating scale to assess denial | 89 coronary patients | Hackett-Cassem Denial Scale |
| Hart et al., 2000 | The relationship between pain and coping styles among HIV-positive men and women | 105 patients with HIV | Coping Orientations to  Problems Experienced |
| Havik and Mæland, 1986 | Dimensions of verbal denial in myocardial infarction: correlates to 3 denial scales | 367 patients with acute myocardial infarction | Havik and Mæland Denial Scale |
| Huntley et al., 2019 | Cardiac denial and expectations associated with depression in adults with congenital heart disease | 78 patients with congenital heart disease | Cardiac Denial of Impact Scale |
| Ironson et al., 1994 | Distress, denial, and low adherence to behavioral interventions predict faster disease progression in gay men infected with human immunodeficiency virus | 23 patients with AIDS | Coping Orientations to Problems Experienced |
| Jacobsen and Lowery, 1992 | Further analysis of the psychometric properties of the Levine Denial of Illness Scale | 152 hospitalized patients with myocardial infarction | Levine Denial of Illness Scale |
| Kamen et al., 2012 | The impact of denial on health-related quality of life in patients with HIV | 65 HIV patients | Coping Orientations to  Problems Experienced |
| Karadere et al., 2019 | Reliability and validity of a Turkih version of the Acceptance and Action Diabetes Questionnaire | 105 endocrinological outpatients | Acceptance and Action Diabetes Questionnaire |
| Kortte et al., 2007 | The hopkins rehabilitation engagement rating scale: development and psychometric properties | 206 patients with spinal cord injury | Levine Denial of Illness Scale |
| Leserman et al., 2000 | Impact of stressful life events, depression, social support, coping, and cortisol on progression to AIDS | 82 patients with HIV type-1 infection without AIDS | Coping Orientations to Problems Experienced |
| Levenson et al., 1984 | Denial predicts favorable outcome in unstable angina pectoris | 26 patients with unstable angina | Hackett-Cassem Denial Scale |
| Levenson et al., 1989 | Denial and medical outcome in unstable angina | 48 patients with unstable angina | Hackett-Cassem Denial Scale |
| Levine et al., 1987 | The role of denial in recovery from coronary heart disease | 45 patients who were hospitalized for myocardial infarction or for coronary bypass surgery | Levine Denial of Illness Scale |
| Levine et al., 1994 | A two factor model of denial of illness: a confirmatory factor analysis | 19 patients with epilepsy, 15 with hypertension, 21 stroke patients, and 45 patients with coronary artery disease | Levine Denial of Illness Scale |
| Mann et al., 2018 | A Canadian survey of self-management strategies and satisfaction with ability to control pain: comparison of community dwelling adults with neuropathic pain versus adults with non-neuropathic chronic pain | 710 chronic pain patients | Coping Orientations to  Problems Experienced |
| McGann et al., 2008 | Denial and compliance in adults with asthma | 51 patients with a diagnosis of asthma | Levine Denial of Illness Scale |
| Nazarian et al., 2006 | A naturalistic study of ambulatory asthma severity and reported avoidant coping styles | 61 patients with a diagnosis of asthma | Coping Orientations to Problems Experienced |
| Noy et al., 1995 | A new approach to affective symptoms in relapsing-remitting multiple sclerosis | 20 consecutive patients with multiple sclerosis | Hackett-Cassem Denial Scale |
| O’Carroll et al., 2001 | Psychological factors associated with delay in attending hospital following a myocardial infarction | 72 myocardial infarction patients | Cardiac Denial of Impact Scale |
| Paredes et al., 2012 | A longitudinal study on emotional adjustment of sarcoma patients: the determinant role of demographic, clinical and coping variables | 36 sarcoma patients | Coping Orientations to  Problems Experienced |
| Perkins-Porras et al., 2008 | Causal beliefs, cardiac denial and pre-hospital delays following the onset of acute coronary syndromes | 177 patients with acute coronary syndrome | Cardiac Denial of Impact scale |
| Picardi et al., 2005 | Psychosomatic assessment of skin diseases in clinical practice | 545 patients with various forms of skin disease | Diagnostic Criteria for Psychosomatic Research |
| Piolanti et al., 2019 | A trial integrating different methods to assess psychosocial problems in primary care | 200 primary care patients | Diagnostic Criteria for Psychosomatic Research |
| Porcelli et al., 2000 | Assessing somatization in functional gastrointestinal disorders: integration of different criteria | 190 patients with functional gastrointestinal disorders | Diagnostic Criteria for Psychosomatic Research |
| Pugi et al., 2022 | Health-related quality of life in pre-dialysis patients with chronic kidney disease: the role of big-five personality traits and illness denial | 100 pre-dialysis patients with chronic kidney disease | Illness Denial Questionnaire |
| Rafanelli et al., 2013 | Psychological correlates of vasovagal versus medically unexplained syncope | 67 patients with suspected vasovagal syncope | Diagnostic Criteria for Psychosomatic Research |
| Rafanelli et al., 2003 | Psychological assessment in cardiac rehabilitation | 61 patients with first myocardial infarction | Diagnostic Criteria for Psychosomatic Research |
| Ramathan-Elion et al., 2016 | The role of psychological facilitators and barriers to therapeutic engagement | 206 patients who were recruited from 3 acute rehabilitation hospitals | Levine Denial of Illness Scale |
| Rossi Ferrario et al., 2017 | Illness denial questionnaire for patients and caregivers | 74 cardiac patients, 49 oncological patients, 48 neurological patients, 35 patients with renal diseases, and 13 with respiratory disorders | Illness Denial Questionnaire |
| Rossi Ferrario et al., 2019 | Development and psychometric properties of a short form of the Illness Denial Questionnaire | 113 patients with various medical conditions | Illness Denial Questionnaire |
| Rossi Ferrario and Panzeri, 2020 | Exploring illness denial of LVAD patients in cardiac rehabilitation and their caregivers: a preliminary study | 32 cardiac patients with implantable left ventricular assist devices | Illness Denial Questionnaire |
| Roussi et al., 2007 | Patterns of coping, flexibility in coping and psychological distress in women diagnosed with breast cancer | 72 patients with breast cancer | Coping Orientations to Problems Experienced |
| Saito et al., 2018 | The reliability and validity for Japanese type 2 diabetes patients of the Japanese version of the acceptance and action diabetes questionnaire | 600 patients with type 2 diabetes | Acceptance and Action Diabetes Questionnaire |
| Santos et al., 2006 | Denial in the first days of acute stroke | 180 consecutive acute stroke patients and a control group of 50 acute coronary patients | Denial of Illness Scale |
| Schmitt et al., 2014 | Assessment of diabetes acceptance can help identify patients with ineffective diabetes self-care and poor diabetes control | 320 patients with type 1 or 2 diabetes | Acceptance and Action Diabetes Questionnaire |
| Sherman et al., 2000 | Coping with head and neck cancer during different phases of treatment | 120 patients with advanced cancer | Coping Orientations to Problems Experienced |
| Spiess et al., 1995 | A program to reduce onset distress in unselected type I diabetic patients: effects on psychological variables and metabolic control | 23 patients with a diagnosis of type I diabetes mellitus | Hackett-Cassem Denial Scale |
| Stenström et al., 2005 | Denial in patients with a first-time myocardial infarction: relations to pre-hospital delay and attendance to a cardiac rehabilitation programme | 107 patients with a first-time myocardial infarction | Hackett-Cassem Denial Scale |
| Tesio et al., 2019 | Utility of the diagnostic criteria for psychosomatic research in assessing psychological disorders in fibromyalgia patients | 98 patients with fibromyalgia and 98 patients with rheumatoid arthritis | Diagnostic Criteria for Psychosomatic Research |
| Trijsburg et al., 1987 | Denial and overcompensation in male patients with myocardial infarction | 122 patients with acute myocardial infarction | Denial Questionnaire |
| Umucu and Lee, 2020 | Examining the impact of COVID-19 on stress and coping strategies in individuals with disabilities and chronic conditions | 269 patients with self-reported disabilities and chronic conditions | Coping Orientations to  Problems Experienced |
| Vos et al., 2007 | The denial of cancer interview: development and first assessment of psychometric properties in lung cancer patients | 195 consecutive newly diagnosed lung cancer patients | Denial of Cancer Interview |
| Vos et al., 2008 | Denial in lung cancer patients: a longitudinal study | 195 newly diagnosed lung cancer patients | Denial of Cancer Interview |
| Vos et al., 2010 | Denial and physical outcomes in lung cancer patients, a longitudinal study | 195 consecutive newly diagnosed lung cancer patients | Denial of Cancer Interview |
| Vos et al., 2011 | Denial and social and emotional outcomes in lung cancer patients: the protective effect of denial | 195 newly diagnosed lung cancer patients | Denial of Cancer Interview |
| Warrenburg et al., 1989 | Defensive coping and blood pressure reactivity in medical patients | 29 cardiac patients | Levine Denial of Illness Scale |
| Weaver et al., 2004 | Perceived stress mediates the effects of coping on the quality of life of HIV-positive women on highly active antiretroviral therapy | 90 patients with HIV | Coping Orientations to  Problems Experienced |
| White et al., 2016 | Cardiac denial and psychological predictors of cardiac care adherence in adults with congenital heart disease | 80 patients with congenital heart disease | Cardiac Denial of Impact Scale |
| Wijk et al., 2024 | Psychometric evaluation of the Swedish Acceptance and Action Diabetes Questionnaire: a Rasch analysis | 120 patients with type 1 diabetes | Acceptance and Action Diabetes Questionnaire |
| Yellowlees and Ruffin, 1989 | Psychological defenses and coping styles in patients following a life-threatening attack of asthma | 25 patients with asthma | Illness Behavior Questionnaire |
